# Sequence Changes Modulate Peptoid Self-Association in Water

**DOI:** 10.3389/fchem.2020.00260

**Published:** 2020-04-23

**Authors:** Amelia A. Fuller, Christian J. Jimenez, Ella K. Martinetto, Jose L. Moreno, Anna L. Calkins, Kalli M. Dowell, Jonathan Huber, Kyra N. McComas, Alberto Ortega

**Affiliations:** Department of Chemistry & Biochemistry, Santa Clara University, Santa Clara, CA, United States

**Keywords:** peptoid, peptidomimetic, self-association, circular dichroism (CD) spectroscopy, fluorescence spectroscopy, size exclusion chromatography

## Abstract

Peptoids, *N*-substituted glycine oligomers, are a class of diverse and sequence-specific peptidomimetics with wide-ranging applications. Advancing the functional repertoire of peptoids to emulate native peptide and protein functions requires engineering peptoids that adopt regular secondary and tertiary structures. An understanding of how changes to peptoid sequence change structural features, particularly in water-soluble systems, is underdeveloped. To address this knowledge gap, five 15-residue water-soluble peptoids that include naphthalene-functionalized side chains were designed, prepared, and subjected to a structural study using a palette of techniques. Peptoid sequence designs were based on a putative amphiphilic helix peptoid bearing structure-promoting (*S*)-*N*-(1-naphthylethyl)glycine residues whose self-association in water has been studied previously. New peptoid variants reported here include sequence changes that influenced peptoid conformational flexibility, functional group patterning (amphiphilicity), and hydrophobicity. Peptoid structures were evaluated and compared using circular dichroism spectroscopy, fluorescence spectroscopy, and size exclusion chromatography. Spectral data confirmed that sequence changes alter peptoids' degree of assembly and the organization of self-assembled structures in aqueous solutions. Insights gained in these studies will inform the design of new water-soluble peptoids with regular structural features, including desirable higher-order (tertiary and quaternary) structural features.

## Introduction

The functions of most proteins rely on their ability to adopt complex folded structures that include intramolecular structural features (secondary or tertiary folds) and, in many cases, well-defined intermolecular contacts (quaternary structure). Researchers wishing to reconstitute proteins' functions, including catalysis and molecular recognition, have sought to develop foldamers, structured synthetic counterparts that mimic structural elements of proteins (Gellman, 1998; David et al., 2001; Goodman et al., 2007; Guichard and Huc, 2011). Peptoids (*N*-substituted glycine oligomers) are highly attractive peptidomimetic foldamer scaffolds for the design of functional molecules with defined three-dimensional structures (Sun and Zuckermann, 2013). Straightforward solid-phase synthesis of peptoids *via* the submonomer approach (Zuckermann et al., 1992) allows them to be prepared sequence-specifically. Diverse peptoid side chain functionality is derived from a rich array of commercially and synthetically available primary amine synthons. Peptoids that adopt regular secondary structures, including helices (Armand et al., 1998; Wu et al., 2001, 2003; Roy et al., 2017; Gimenez et al., 2018, 2019), sheets (Crapster et al., 2011), and other regular structures (Gorske et al., 2017) have been described and applied. The origin of peptoids' three-dimensional structures is backbone organization by controlling the energetics of the *cis/trans*-amide configurations. Stereoelectronic features of the appended *N*-substituents influence this conformational isomerization (Gorske et al., 2009). For example, oligomers with sterically bulky and/or electron-poor *N*-substituents generally favor the *cis*-amide bond (Gorske et al., 2009); this configuration promotes a peptoid helix secondary structure with three residues per turn and a helix pitch of 6 Å (Armand et al., 1998). Investigations into the contributions of both specific side chains and sequence ordering to peptoid secondary structure have begun to clarify sequence requirements for the predictive design of peptoids with helical structures (Wu et al., 2001; Shin et al., 2014).

Two significant challenges hamper the development of functional peptoids that rival the structural complexity of proteins. First, a number of putative applications for peptoids with complex structures, including those in biological systems, will necessitate water-soluble peptoids. Thus, a deeper understanding of peptoid structure in aqueous solution is needed to complement the larger body of work evaluating peptoid structures in organic solution (Armand et al., 1998; Wu et al., 2001, 2003; Stringer et al., 2011; Shin et al., 2014). Recently, a number of researchers have developed strategies to prepare peptoids that are structured and functional in aqueous solution (Sanborn et al., 2002; Shin and Kirshenbaum, 2007; Baskin and Maayan, 2015; Gimenez et al., 2018, 2019). Similarly, water-soluble versions of structured β-peptoid helices, a closely related oligomer, have recently been reported (Wellhöfer et al., 2019). Second, there are relatively few examples of sequence-specific, water-soluble peptoids that adopt ordered tertiary or quaternary structures. These include helix bundles (Burkoth et al., 2002; Byoung-Chul et al., 2005; Lee et al., 2008; Fuller et al., 2013b), nanosheets (Nam et al., 2010; Robertson et al., 2016), superhelices (Murnen et al., 2010), and membrane mimics (Jin et al., 2016). The paucity of examples of peptoids that approach the structural complexity demonstrated by proteins underscores our incomplete understanding of sequence features that mediate peptoid self-association.

**Figure 1:**
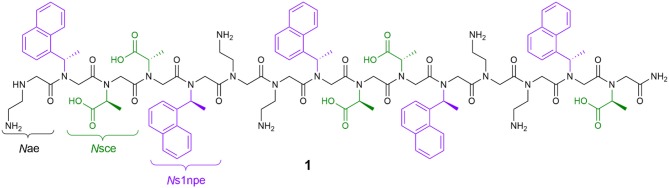
Structure of peptoid **1**, a 15-residue, water-soluble peptoid with putative amphiphilic helix structure that self-associates in aqueous solution.

In our own work, we have detailed the length- and solvent-dependent self-association of peptoid **1**, a water-soluble, 15-residue, putative amphiphilic helix (Figure 1) (Fuller et al., 2013b, 2018a,b). Given the challenges of identifying and studying peptoids with higher-order structures, the self-association of **1** gives us insight into features that enable this desired structural complexity. The sequence of **1** includes only three unique residues: *N*-(2-aminoethyl)glycine (*N*ae, positively charged at neutral pH), (S)-*N*-(1-carboxyethyl)glycine (*N*sce, negatively charged at neutral pH), and (S)-*N*-(1-naphthylethyl)glycine (*N*s1npe, aromatic and hydrophobic). Importantly, the *N*s1npe residue is helix-promoting because it favors strongly the *cis*-amide conformation (Gorske et al., 2009). The patterning of these residues in the sequence of **1** is expected to arrange all of the aromatic *N*s1npe side chains on the same face of the helix. The self-association of this molecule imparts **1** with properties that could translate to interesting potential applications, including molecular recognition of small organic molecules (Fuller et al., 2018b).

To expand the applications of **1** and similar peptoids, we explored the effects of peptoid sequence changes on self-association. Here, we report the design and synthesis of five peptoids: **1** and four sequence variants of **1** that modulate peptoid secondary structure, amphiphilicity, and hydrophobicity; we hypothesized that all of these features may contribute to peptoid secondary structure and self-association. These five peptoids were studied using a palette of techniques to evaluate their secondary structures as well as their ability to form aggregates. From the data, we identified how these sequence attributes—conformational flexibility, sequence patterning, and hydrophobicity—contribute to peptoid self-association. Results from these studies add to our understanding of sequence–structure relationships in this important class of peptidomimetic and will inform the design of new, functional peptoids.

## Materials and Methods

### Materials

Reagents were purchased from commercial suppliers and used without further purification. 4-Methylpiperidine, bromoacetic acid, trifluoracetic acid, and triisopropylsilane were purchased from ACROS. Diisopropylcarbodiimide (DIC) was purchased from Anaspec. (S)-(–)-1-(1-naphthyl)ethylamine (used to generate the *N*s1npe residue), (S)-(–)-1-(2-naphthyl)ethylamine (used to generate the *N*s2npe residue), and (S)-(–)α-methylbenzylamine (used to generate the *N*spe residue) were purchased from Aldrich. Alanine-*tert*-butyl ester hydrochloride (used to generate the *N*sce residue) was purchased from AAPPTec (Louisville, KY). Before use, the ammonium salt was dissolved in a minimal amount of water, and 2.5 M sodium hydroxide (NaOH) was added to adjust the solution pH to 10. The neutralized amine was then extracted with dichloromethane (CH_2_Cl_2_), and the organic solution was dried over MgSO_4_ then concentrated by rotary evaporation. *tert*-Butyl *N*-(2-aminoethyl)carbamate (used to generate the *N*ae residue) was purchased from Oakwood Chemical. Fmoc-protected Rink amide resin (0.74 or 0.38 mmol/g) was purchased from Novabiochem (San Diego, CA). Solvents for peptoid synthesis, *N, N'*-dimethylformamide (DMF) and *N*-methyl-2-pyrrolidone (NMP), were purchased from Fisher Scientific. 1-Anilinonaphthalene-8-sulfonic acid (1,8-ANS) was purchased from Aldrich. Guanidinium hydrochloride and ammonium sulfate were purchased from Fisher Scientific. Buffers were prepared by diluting the appropriate solute into ultrapure water and adjusting pH by addition of concentrated NaOH or sulfuric acid (H_2_SO_4_) solutions. Tris-buffered saline (TBS buffer) is 50 mM Tris, 150 mM NaCl, pH 7.5.

### Peptoid Synthesis and Purification

Peptoids were prepared on solid support following adaptations to previously reported procedures (Zuckermann et al., 1992). Rink amide resin (0.05 mmol) was added to a 25-ml glass vessel fitted with a coarse glass frit and a Teflon stopcock and rinsed twice with CH_2_Cl_2_. The resin was then allowed to swell in 1.5 ml DMF for 30 min. The DMF was drained, 2.5 ml of a 20% 4-methylpiperidine solution in DMF was added to the resin, and the capped vessel was agitated on a wrist-action shaker for 10 min at room temperature. This deprotection reaction was repeated, and the resin was rinsed with DMF (7 × 2 ml).

Peptoids were prepared sequence-specifically by alternate reactions with bromoacetic acid/DIC and with the appropriate primary amine. Bromoacetylation was effected by adding bromoacetic acid (830 μl of a 1.2-M solution in DMF, 19.9 equiv.) and DIC (200 μl, 25.5 equiv.) to the resin. The mixture was agitated on a wrist-action shaker for 20 min, drained, and washed with DMF (7 × 2 ml). The appropriate primary amine (1 ml of a 1-M solution in NMP, 20 equiv.) was then added to the reaction vessel, and the mixture was agitated by shaking for 40 min. For the installation of the *N*s1npe, *N*npm, or *N*s2npe residues, (*S*)-1-(1-naphthyl)ethylamine, 1-napthylmethylamine, or (S)-1-(2-naphthyl)ethylamine) was added, respectively. The *N*ae and *N*sce residues were installed by the addition of *tert*-butyl *N*-(2-aminoethyl)carbamate, alanine *tert*-butyl ester, respectively. After each amination reaction, the solution was drained, washed five times with DMF then five times with CH_2_Cl_2_. Progress of the reactions was monitored using the acetaldehyde/chloranil test; blue/green beads indicated the presence of a secondary amine. Following the final amination reaction, peptoids were cleaved from the resin by equilibration with 2 ml trifluoroacetic acid/H_2_O/triisopropylsilane (95:2.5:2.5) for 2 h. The filtrate was collected, and the resin was washed with methanol (2 × 2 ml). The filtrate and methanol washes were combined, then concentrated by rotary evaporation.

Crude peptoids were purified by reverse-phase high-performance liquid chromatography (RP-HPLC) on a Hitachi Chromaster 5000 instrument equipped with a semi-preparative AAPPTec Spirit C18 column (5 mM, 10.0 mm × 25 cm) using a linear gradient of 30–90% methanol (solvent B) in 0.1% aqueous trifluoroacetic acid (TFA; solvent A) at a flow rate of 3 ml/min. Peaks eluted were detected by absorbance at 220 nm. All data were visualized with EZChrom software. The purity of the synthetic peptoids was assessed by analytical HPLC (see Supplementary Material).

Purified peptoids were lyophilized to afford white powders. The molar masses of purified peptoids were confirmed by electrospray mass spectrometry in positive ion mode using a Thermo LCQ Fleet mass spectrometer. High-resolution mass spectral data for new compounds were acquired on an Agilent QTOF6520 mass spectrometer (data in Supplementary Material).

### Preparation of Peptoid Solutions for Spectroscopy Studies

Concentrated stock solutions of peptoids (≥1 mM) were prepared by dissolving the lyophilized peptoid in methanol. Measurement of solutions' UV absorption enabled calculation of the solutions' concentrations [ε = 13,300 M^−1^ cm^−1^ at for **1–3** at 266 nm; ε = 6,141 M^−1^ cm^−1^ at for **4** at 266 nm; ε = 5,635 M^−1^ cm^−1^ at for **5** at 266 nm; all molar absorptivities were measured in phosphate buffered saline (PBS) buffer]. Solutions for spectroscopy studies were made by dilution of the concentrated stock into the appropriate buffer or solvent. For aqueous solutions, the solutions analyzed were ≤ 10% methanol by volume.

### Circular Dichroism Spectroscopy

Circular dichroism (CD) spectra were acquired using an OLIS rapid scanning monochromator (RSM) equipped with a Quantum TC 125 temperature controller. Spectra were collected from 190 to 320 nm in 0.5-nm increments. Data were averaged for 5 s at each wavelength. Data were originally collected in millidegrees and then converted to per-residue molar ellipticity (deg cm^2^/dmol). A spectrum of the solvent was subtracted from peptoid solution spectra. Variable-temperature CD spectra were collected for peptoids as detailed in the *Results and Discussion*. Spectra were collected from 200 to 260 nm at 0.5-nm increments, and data were averaged for 5 s at each wavelength. Spectra were taken at 2, 20, 40, 60, 80, and 98°C with an equilibration time of 2 min at each temperature. For each peptoid, spectra were collected upon both increasing temperatures and decreasing temperatures. Buffers used for these experiments were: 5 mM Tris, pH 7.4; 10 mM phosphate + 6 M guanidinium hydrochloride, pH 7.5; 10 mM phosphate + 3 M ammonium sulfate, pH 7.5.

### Fluorescence Spectroscopy

All fluorescence emission spectra were acquired on a Molecular Devices SpectraMax i3x plate reader instrument, and experimental setup and data extraction were done with SoftMax Pro 6.5.1. Solutions for analysis were distributed into a black, flat-bottom, 96 standard well opaque plate (150 μl/well). Each solution was added to three wells, and results reported are the average of data collected for these three replicates. Excitation and emission slit widths were set to 9 and 15 nm, respectively.

#### Peptoid Fluorescence Spectra

The excitation wavelength was set to 270 nm. Emission spectra of peptoids at 5 and 100 μM were collected from 300 to 500 nm in 1-nm increments. Buffers used for these experiments were: 5 mM Tris buffer, pH 7.4; 10 mM phosphate + 6 M guanidinium hydrochloride, pH 7.5; 10 mM phosphate + 3 M ammonium sulfate, pH 7.5.

#### 1-Anilinonaphthalene-8-Sulfonic Acid Fluorescence Spectra

Fluorescence emission spectra of 50 mM 1,8-ANS were acquired in the presence of [peptoid] = 0, 10, 25, 50, 100 μM in the specified buffer. The excitation wavelength was set to 380 nm, and emission spectra were collected from 420 to 700 nm in 1-nm increments. Buffers used for these experiments were: TBS; 10 mM phosphate + 6 M guanidinium hydrochloride, pH 7.5; 10 mM phosphate + 3 M ammonium sulfate, pH 7.5.

### Size Exclusion Chromatography

Size exclusion chromatography (SEC) was carried out on an AKTA liquid chromatographic system equipped with a Superdex Increase GL 10/300 column (GE Healthcare). The system was kept at 4°C during all experiments. A 200-μl sample of peptoid at a concentration of 500 μM in the appropriate buffer (TBS, 10 mM phosphate + 6 M guanidinium hydrochloride, or 10 mM phosphate + 3 M ammonium sulfate buffers) were loaded onto a 1-ml injection loop and was injected and eluted from the column in the same buffer at a flow rate of 0.5 ml/min. In separate injections, molecular weight standards were also eluted with TBS buffer at 0.5 ml/min, and peaks were detected by absorbance at 280 nm. Standards injected were: cytochrome C (M_r_ = 12,384), aprotinin (M_r_ = 6,512), vitamin B12 (M_r_ = 1,855), cytidine (M_r_ = 243). A plot of log M_r_ vs. retention time was generated from the elution of the standards. The observed molecular weight of each peptoid analyzed in a given elution solution was interpolated from this line. The degree of assembly was calculated by dividing the observed molecular weight by the molecular weight of the peptoid. Chromatograms are included in Supplementary Material.

## Results and Discussion

### Peptoid Design and Synthesis

**Figure 2:**
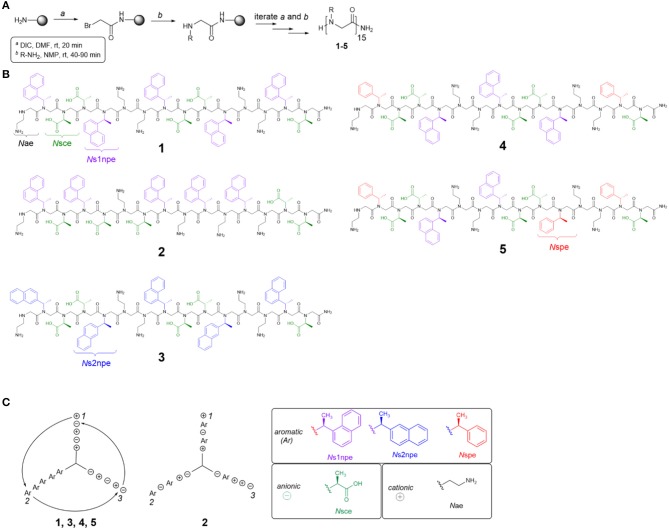
Peptoid synthesis and structures used in this study. **(A)** Peptoid synthesis following the submonomer method. DIC, diisopropylcarbodiimide. **(B)** Structures of all peptoids prepared. **(C)** Helix wheel representations of peptoids studied. Ar, aromatic residue (*N*s1npe, *N*s2npe, or *N*spe); *N*sce, negative charge; *N*ae, positive charge.

To evaluate the contributions of conformational flexibility, sequence patterning (amphiphilicity), and hydrophobicity to peptoid structure and self-association in aqueous solutions, the previously studied **1** and four new variants (**2–5**) were designed and prepared (Figure 2, Table 1). All peptoids were prepared sequence-specifically on an amine-functionalized resin following the submonomer method (Figure 2A) (Zuckermann et al., 1992). Individual residues were installed by iterating bromoacetylation reactions followed by displacement of the bromine with the desired primary amine. Crude peptoids were subsequently purified by RP-HPLC and identified by mass spectrometry (see Supplementary Material).

**Table 1:** Sequences of peptoids studied.

**Peptoid**	**Monomer sequence (*N*-terminus to *C*-terminus)**
**1**	*N*ae-Ns1npe-(Nsce)_2_-Ns1npe-(*N*ae)_2_-Ns1npe-(Nsce)_2_-Ns1npe-(*N*ae)_2_-Ns1npe-Nsce *previously studied helical, amphiphilic sequence*
**2**	*N*ae-Ns1npe- Nsce- Ns1npe-Nsce-*N*ae- Nsce-Ns1npe-*N*ae-Ns1npe-*N*ae-Ns1npe-*N*ae-(Nsce)_2_ *non-amphiphilic sequence patterning*
**3**	*N*ae-*N*s2npe-(Nsce)_2_-*N*s2npe-(*N*ae)_2_-*N*s2npe-(Nsce)_2_-*N*s2npe-(*N*ae)_2_-*N*s2npe-Nsce *modulates secondary structure by lowering K_*cis*/*trans*_*
**4**	*N*ae-Nspe-(Nsce)_2_-Ns1npe-(*N*ae)_2_-Ns1npe-(Nsce)_2_-Ns1npe-(*N*ae)_2_-Nspe-Nsce *modulates secondary structure by lowering K_*cis*/*trans*_ and*
	*decreases hydrophobicity*
**5**	*N*ae- Nspe-(Nsce)_2_-Ns1npe-(*N*ae)_2_-Ns1npe-(Nsce)_2_-Nspe-(*N*ae)_2_-Nspe-Nsce *modulates secondary structure by lowering K_*cis*/*trans*_ and*
	*decreases hydrophobicity*

*Below each sequence is noted the anticipated effects of sequence changes relative to previously studied peptoid **1**. Colors of residues match colors in the structures shown in Figure 2*.

Five different residues were incorporated into peptoids **1–5** (Figure 2B, Table 1). Polar residues, which impart significant water solubility, were the same in all sequences: the cationic *N*ae and the anionic *N*sce. Between the sequences, aromatic residues, *N*s1npe, (S)-*N*-(1-naphthylethyl)glycine (*N*s2npe), and (*S*)-*N*-(1-phenylethyl)glycine (*N*spe), were varied to modulate peptoid conformational flexibility and hydrophobicity (Table 1). We reasoned that either flexibility or hydrophobicity could influence peptoids' ability to self-associate as well as the structures of their aggregates. Further, we wished to examine the importance of sequence patterning on peptoid structure and self-association. Peptoid **2** was designed to be a non-amphiphilic isomer of **1**. As seen in the helix wheel representations (Figure 2C), peptoid **2** arranged the aromatic residues on all three faces of the putative helix structure. All of the other peptoids maintained the predicted amphiphilic arrangement of residues.

Peptoid conformational flexibility was related to heterogeneity in *cis/trans*-amide bond configurations; residues that favor the *cis*-amide bond (i.e., have higher *K*_*cis*/*trans*_) promote the helix structure (Armand et al., 1997). The Blackwell lab determined *K*_*cis*/*trans*_ for a variety of model compounds (Gorske et al., 2009). From this work, it was predicted that *N*s1npe most strongly promotes the helix [*K*_*cis*/*trans*_ of model analog = 6.27 in trideuteroacetonitrile (CD_3_CN)] (Gorske et al., 2009; Stringer et al., 2011). The other two aromatic residues, *N*spe and *N*s2npe, have lower energetic preference for the *cis*-amide configuration: *K*_*cis*/*trans*_ of the corresponding model compounds = 2.04 and 2.21, respectively, in CD_3_CN (Gorske et al., 2009). As such, inclusion of *N*spe and *N*s2Npe residues was expected to introduce more conformational flexibility into the peptoid. Peptoid **3** substitutes the *N*s2npe residue for the *N*s1npe residues in **1**. This change was anticipated to generate a more conformationally flexible but still amphiphilic helix. Peptoids **4** and **5** likewise maintain the amphiphilic sequence patterning but substitute two or three of the *N*s1npe aromatic residues in **1** for the less helix-promoting *N*spe residue.

Peptoid hydrophobicity was also adjusted by substituting *N*s1npe residues in **1** with the smaller *N*spe residues (peptoids **4** and **5**). Indeed, RP-HPLC retention times of **4** and **5** were lower than retention times of **1–3** (Supplemental Material). These comparisons were consistent with the prediction that substituting the *N*spe residue for *N*s1npe generates a less hydrophobic peptoid.

### Peptoids 1–5 Have Different Elution Times in Size Exclusion Chromatography

Initial evidence for the ability of **1–5** to self-associate in aqueous solution was sought using SEC. Peptoid solutions at a concentration of 500 μM were prepared for this analysis, and this solution was diluted at least 5-fold upon injection. We have previously reported that **1** elutes with an apparent molecular weight over twice the unassociated peptoid mass (Fuller et al., 2013b, 2018a). We compared the retention times for solutions of our new peptoids, **2–5**, to retention times of molecular weight standards to estimate the apparent molecular weights of the peptoids in solution (Table 2). Notably, the non-amphiphilic **2** was not sufficiently soluble in TBS at the high solution concentrations needed for this analysis. This was our first evidence that **2** can aggregate in aqueous solution. The predicted irregular display of hydrophobic and polar groups in its helix structure apparently contributed to the formation of insoluble aggregates. Peptoid **3** eluted from the column with an apparent molecular weight four times its monomeric molecular weight, suggesting it self-associates in a neutral aqueous buffer to an even greater extent than **1**. Interestingly, **4** also eluted from the column with an observed molecular weight that was also twice its monomeric molecular weight. Peptoid **5**, with only two naphthalene chromophores, was challenging to evaluate because it gave low absorbance at the detection wavelength (280 nm).

**Table 2:** Estimated assembly sizes of peptoids as determined by SEC.

**Peptoid**	**Observed molecular weight**	**Degree of assembly^a^**
**1**	5,022	2.3
**2**	ND	ND
**3**	8,560	4.0
**4**	4,611	2.1
**5**	ND	ND

a *Degree of assembly = observed molecular weight from SEC experiment/peptoid molecular weight. ND, not determined (see text); SEC, size exclusion chromatography*.

Together, these data indicated that a range of peptoids with naphthalene-functionalized side chains could self-associate in neutral, aqueous solution. The insolubility of **2** at high concentrations and the high observed molecular weight of **3** suggested that either additional conformational flexibility or changes to sequence patterning could promote peptoid self-association. The observed molecular weight of **4** clarified that amphiphilic peptoid sequences with smaller aromatic residues may also self-associate.

### Peptoids Influence the Fluorescence Emission of 1-Anilinonaphthalene-8-Sulfonic Acid

**Figure 3:**
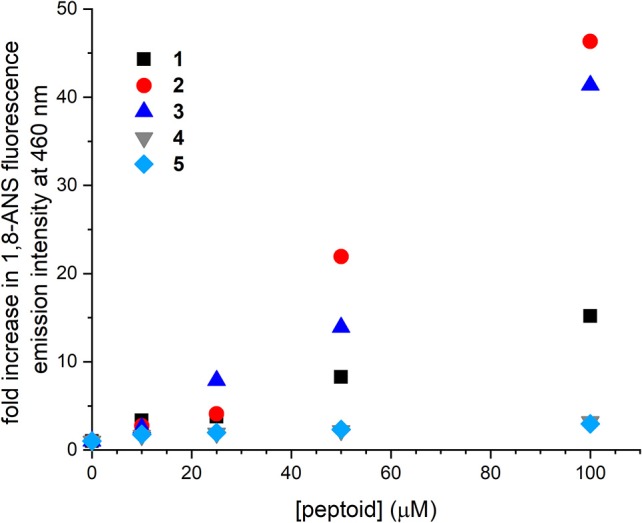
Changes to fluorescence emission intensity of 1-anilinonaphthalene-8-sulfonic acid (1,8-ANS) as influenced by increasing concentrations of **1–5**. Spectra were collected for solutions of 50 μM 1,8-ANS plus varied concentrations of peptoid in Tris-buffered saline (TBS buffer) at room temperature.

The interactions of **1–5** with the environmentally sensitive fluorophore 1,8-ANS were evaluated as a complementary approach to explore the self-association of these sequences (Figure 3). 1,8-ANS is an environmentally sensitive fluorophore, and its interaction with hydrophobic microenvironments causes enhanced emission intensity and blue-shifting of its emission λ_max_ (Semisotnov et al., 1991). 1,8-ANS and similar fluorophores have been used to evaluate peptoid self-association and folding because they provide a sensitive readout for hydrophobic surfaces that may be formed upon aggregation (Burkoth et al., 2002; Murnen et al., 2012; Fuller et al., 2013a, 2018a). Similarly, emission of 1,8-ANS has been used to monitor protein unfolding; exposure of the hydrophobic interior of the protein correlates with enhanced fluorophore emission (Hawe et al., 2008).

Changes to fluorescence emission of 1,8-ANS were consistent with the apparent molecular weights for **1–3** observed by SEC. As previously detailed, increasing concentration of **1** to 100 μM increased 1,8-ANS fluorescence intensity by 15-fold, consistent with self-association of **1** (Fuller et al., 2013b, 2018a). In the presence of 100 μM **2** and **3**, 1,8-ANS fluorescence emission was increased 46 and 41-fold, respectively. The greater emission intensity of 1,8-ANS in the presence of **2** and **3** compared to **1** suggested that **2** and **3** form larger and/or more hydrophobic surfaces that are accessible to the dye. These hydrophobic patches could be solvent-exposed or in buried areas within a larger aggregate that sequester the 1,8-ANS from the aqueous solvent. Taken together with the low solubility of **2** at high concentrations and the observed molecular weight of **3** by SEC, the experimental data supported that **2** and **3** self-associate to form larger aggregates than **1** can. From these experiments, we learned that peptoids with altered sequence patterning and/or conformational heterogeneity may self-associate in aqueous solution.

Increasing concentrations of **4** and **5** influenced the emission intensity of 1,8-ANS much less than the other peptoids. At 100 μM **4** or **5**, the emission intensity of the dye was increased by only about 3-fold. We considered two possible origins of this observation. First, **4** and **5** may not self-associate to the same extent as the other peptoids. Second, because they are less hydrophobic, they may not have as strong an effect on 1,8-ANS fluorescence emission, even if they were self-associating to the same extent. The increase in the dye's emission intensity over the concentration range coupled with the observed molecular weight of **4** in the SEC study were consistent with at least some self-association of each of these peptoids.

The interpretation of these data necessitated that we also consider the effects of ordering in the putative self-associated structures. 1,8-ANS will only interact with hydrophobic surfaces that are sufficiently solvent-exposed or otherwise accessible to the dye (Hawe et al., 2008). Indeed, absence of 1,8-ANS binding was used to verify a well-ordered assembly of β-peptide helices, for example (Goodman et al., 2008). As such, we considered that aggregates of **2** or **3** might not just be larger but may also (or instead) be less well-ordered than aggregates of **1**, **4**, and **5**.

### Different Circular Dichroism Spectral Features Suggest Different Naphthalene Ordering in 1–5

**Figure 4:**
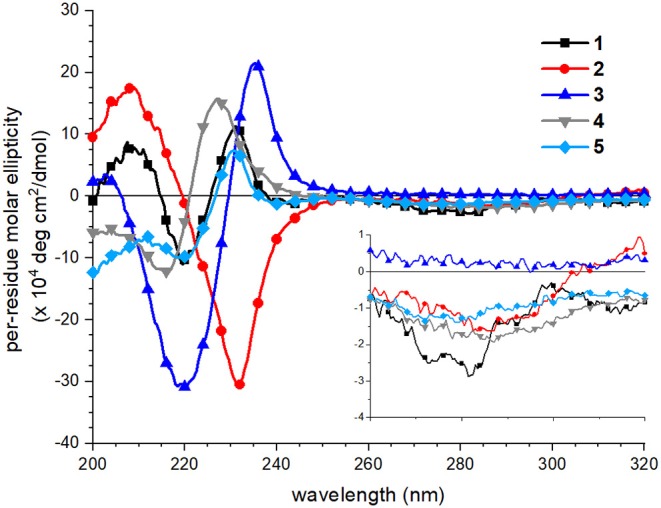
Circular dichroism (CD) spectra of peptoids **1** (black squares), **2** (red circles), **3** (blue triangles, point up), **4** (gray triangles, point down), **5** (light blue diamonds). Peptoids were 40 μM in 5 mM Tris buffer, pH 7.5, and spectra were acquired at 20°C. Inset shows the near-UV region of the spectrum (260–320 nm).

CD spectra of peptoids **1–5** in neutral aqueous buffer solution were acquired to evaluate how sequence changes influenced peptoid structure. CD spectroscopy has routinely been used to evaluate and compare peptoids' secondary structures in solution (Wu et al., 2001, 2003; Sanborn et al., 2002; Shin and Kirshenbaum, 2007; Maayan et al., 2009; Stringer et al., 2011; Shin et al., 2014; Gimenez et al., 2018). In buffer at neutral pH, all five peptoids showed distinct spectral features, particularly in the far-UV region of the spectrum (200–260 nm; Figure 4). As has been previously reported, the far-UV CD spectrum of **1** included maxima at 209 and 231 nm and a minimum at 220 nm (Fuller et al., 2013b, 2018a). The split minimum/maximum at 220 and 231 nm was consistent with exciton-coupled circular dichroism (ECCD) of the naphthalene ^1^B_b_ absorption band. ECCD features indicated that there was regular ordering of the naphthalene chromophores (Sisido et al., 1983a,b; Sisido and Imanishi, 1986; Shoji et al., 2002), consistent with the predicted amphiphilic helix structure wherein all naphthalenes are arranged on the same helix face. Other well-structured naphthalene-functionalized oligomers or polymers also exhibit ECCD spectral features (Sisido et al., 1983a,b; Sisido and Imanishi, 1986; Shoji et al., 2002). The far-UV CD spectrum of **2**, which has different sequence patterning from **1**, shared the maximum at 209 nm, but included a single minimum at 231 nm. The lack of the ECCD signature suggested that ECCD was correlated with the arrangement of *N*s1npe residues on the same helix face. An ECCD signal was observed in the far-UV CD spectrum of **3** with a split minimum/maximum at 220 and 235 nm, respectively. The ECCD for **3** likewise suggested an ordered arrangement of the naphthalene chromophores in this helix, despite the expected flexibility of the amide backbone in **3** relative to the backbone of **1**. The far-UV CD spectra of **4** and **5** included side-chain contributions from both the *N*spe and the *N*s1npe residues. They approached minima at 200 nm; this spectral feature is commonly attributed to π-π^*^ transitions for *N*spe residues (Wu et al., 2001). Both **4** and **5** showed ECCD in the naphthalene absorbance range, although the specific wavelengths of their respective minima/maxima differ slightly. The spectrum of **4** had a minimum at 217 and maximum at 227 nm, while the spectrum of **5** had a minimum at 220 nm and a maximum at 231 nm, analogous to the spectrum of **1**. For all peptoids, CD spectra in methanol did not exhibit ECCD (see spectra in Supplementary Material). This observation was consistent with spectra of poly(*N*s1npe) oligomers previously studied in organic solution (Stringer et al., 2011). Further, it highlighted that naphthalene side chain ordering is a structural feature of peptoids only when they are in aqueous solution.

Spectral features in the near-UV region (260–320 nm), attributed to the naphthalene ^1^L_a_ absorbance, were less sensitive to sequence changes. Although **1** exhibited a weak bisignate ECCD signal in this spectral region, absorbance of the other peptoids was minimal. This observation suggested that the naphthalene chromophores in peptoids **2–5** were less well-organized than they were in **1**.

### Circular Dichroism Spectral Features of 1–5 Are Susceptible to Thermally Induced Changes

**Figure 5:**
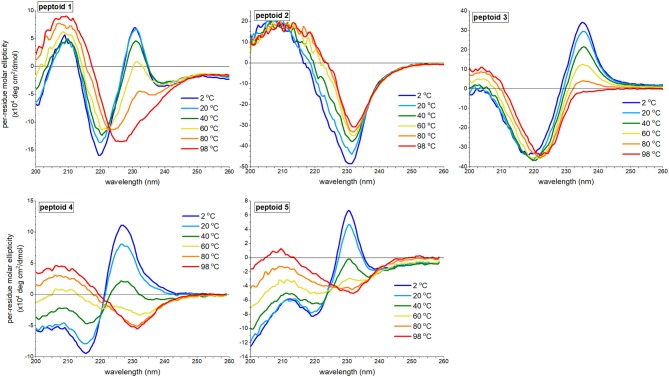
Far-UV circular dichroism (CD) spectra of **1–5** at varied temperatures ranging from 2 to 98°C. All peptoids were 50 μM in 5 mM Tris buffer, pH 7.5.

We reasoned that peptoid structure, including structure attributed to self-association, would be modulated by temperature. We investigated the variation of aqueous far-UV CD spectral features of **1–5** (Figure 5). Previously, we reported that increasing temperatures depletes the absorbance of **1** at both the minimum at 220 nm and the maximum at 231 nm and increases the absorbance at 209 nm; the spectral features of **1** at 98°C resemble the spectrum of **1** in methanol, and no thermal hysteresis was observed (Fuller et al., 2013b). The signal at 231 nm had a non-linear transition over the temperature range monitored, though does not exhibit an inflection point (see Supplementary Material), and there is an isodichroic point at 223 nm. These features are commonly correlated with a two-state transition. The changes to CD spectra of peptoids **4** and **5** bore similarities to the thermally induced changes of the CD spectra of **1**. There was a loss of the ECCD signal, and the CD signal change at 231 nm as a function of temperature was non-linear, including an inflection point (see Supplementary Material). There is not a perfect isodichroic point for either **4** or **5**, but CD signals are very similar at 221 nm for **4** and at 225 nm for **5** at all temperatures measured.

By contrast, the spectra of peptoids **2** and **3** exhibited very different temperature responses from those of **1**. In the CD spectrum of **2**, changes were modest compared to those observed for **1**; the intensity of the minimum at 231 nm decreased, and the minimum red-shifted slightly as the temperature increases. Some thermal hysteresis was observed (see Supplementary Material), and there was no isodichroic point. There were also large changes to the spectral features of **3** upon heating: the maximum at 235 was eliminated, the minimum red-shifted to 222 nm, and the intensity of the maximum at 203 nm increased. The change in signal intensity at 235 nm as a function of temperature was near linear (see Supplementary Material), and there was no isodichroic point for the transition.

We recognized that temperature increases could influence a peptoid's self-association and/or its secondary structure. We posited that temperature-induced spectral changes in **1** were largely attributed to changes in self-association; higher temperatures induced intermolecular dissociation, rather than intramolecular secondary structure changes. The temperature response of **1** was comparable to spectral trends observed for a β-peptoid polymer functionalized with (*S*)-1-naphthylethyl side chains (Laursen et al., 2015). In the β-peptoid, these changes were not correlated with a loss of secondary structure. In contrast, we attributed the temperature-induced loss of ECCD in the spectrum of peptoid **3** to conformational rearrangement. Owing to the lower *K*_*cis*/*trans*_ for the *N*s2npe residues than the *K*_*cis*/*trans*_ for the *N*s1npe residues of **1** (Gorske et al., 2009), a lower energy was required to induce conformational rearrangement. However, we could not rule out a coupled dissociation-unfolding transition of **3** upon heating. The spectra of **2**, which do not include ECCD signals, were minimally changed by temperature. We suggested that loss of CD signal intensity at elevated temperatures was attributed to a modest increase in structural heterogeneity of **2**. We posited that heating **4** and **5** induced both dissociation and changes to peptoid secondary structure.

### Fluorescence Spectral Features of 1–5 Are Concentration-Dependent

**Figure 6:**
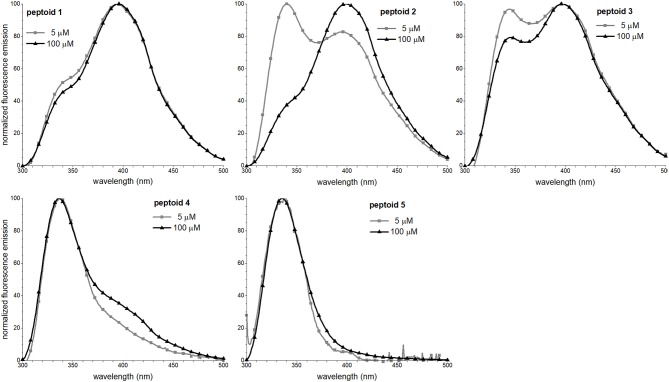
Fluorescence emission spectra of **1–5**. Spectra were recorded at room temperature for peptoid solutions in 5 mM Tris buffer, pH 7.5.

We leveraged the intrinsic fluorescence of the naphthalene chromophore to examine the structure in **1–5**. We have shown in prior work that the fluorescence emission spectrum of **1** exhibited an excimer band (Fuller et al., 2013b, 2018a). The excimer emission results from relaxation of an excited state dimer of naphthalenes, and chromophores must be quite close in space (typically, 4 Å) (Förster, 1969). In similar oligomers and polymers functionalized with naphthalenes, excimer emission has been correlated with both aggregation and conformational heterogeneity; in other words, both inter- and intramolecular interactions may contribute to excimer intensity (Sisido et al., 1983a; Machi et al., 2006; Jimenez et al., 2019). Notably, the excimer intensity of **1** increases with an increase in peptoid concentration, suggesting that self-association contributed to excimer formation. To explore the concentration dependence of the fluorescence emission spectral features of **1–5**, we compared the spectra at 5 and 100 μM peptoid (Figure 6). Except for **5**, an increase in concentration from 5 to 100 μM increased the relative intensity of the peptoid excimer emission band. For **1**, the change is rather modest, suggesting that structure of **1**, or at least the environment of the naphthalenes in the side chains, changed little over this concentration range. In contrast, the emission spectrum of **2** changed more dramatically. At 5 μM of the non-amphiphilic **2**, the excimer emission was lower than the naphthalene monomer emission, consistent with more limited self-association. We speculated that amphiphilic sequence patterning enhanced self-association at low concentrations. At 100 μM, the excimer/monomer emission ratio for **2** was higher than it was for **1**. Attributing excimer emission here to self-association was consistent with the observed low solubility of **2** at high concentration. Peptoid **3** exhibited an enhancement in the relative intensity of the excimer band as peptoid concentration increased, also consistent with its self-association. Spectral changes were more modest for **4**. Because the sequence of **4** has fewer naphthalene-functionalized residues, we could not confidently evaluate if the low intensity of excimer was attributed to a low degree of self-association. In the case of **4** or **5**, we could not rule out that aggregates of these peptoids may exist (as suggested by SEC for **4**), but their structures do not situate naphthalenes sufficiently close to one another to enable efficient excimer emission. In methanol, all peptoids exhibited only the monomer emission (Supplementary Material).

### Chaotrope and Kosmotrope Additives Influence Solution Behavior of 1–5

We reasoned that comparisons of peptoids' solution behavior, including their spectral features, in the presence of solvent additives would further clarify self-association behavior of different sequences. In analogous studies of aggregating peptides, chaotrope additives (e.g., guanidinium hydrochloride) are commonly used to disrupt aggregation while kosmotrope solution additives (e.g., ammonium sulfate) are used to promote hydrophobic interactions and aggregation (Zhang and Cremer, 2006; Buettner et al., 2017). An understanding of the effects of chaotropes and kosmotropes on peptoid structures or other foldamers is not well-developed. In our earlier work with **1**, we observed that 6 M guanidinum hydrochloride promoted its self-assembly (Fuller et al., 2018a). Further, we speculated that the structure of **1** becomes more ordered in this solvent owing to increased solubilization of hydrophobic groups in less-organized assemblies. In ammonium sulfate, however, we suggested that aggregates of **1** were less ordered than in the absence of this additive.

We first undertook SEC analysis of **1–4** in solutions of 6 M guanidinium hydrochloride and 3 M ammonium sulfate. Notably, none of the peptoids was sufficiently soluble at the high concentrations needed for these analyses in the ammonium sulfate solution. Peptoids were all soluble in 6 M guanidinium hydrochloride, and the observed molecular weights of **1** and **4** in this solvent were higher than they were in TBS buffer (see chromatograms and data in Supplementary Material). These results were not surprising given our earlier work with **1** (Fuller et al., 2018a). We speculated that the chaotrope additive better solubilized larger molecular weight aggregates of **1** and **4**. Alternatively (or additionally), we theorized that the high ionic strength of the chaotrope solution might be responsible for promoting hydrophobic interactions and thereby promote aggregation. Another effect we considered is that the high concentration of chaotrope additive screens charges, including the *N*-terminal positive charge, allowing peptoids to self-associate in varied orientations (e.g., parallel or antiparallel). An analogous entropic stabilization of aggregates has been observed in self-associating peptides (Do et al., 2013). Peptoids **2** and **3** also eluted as apparent oligomers in this solvent with degrees of assembly near 3.

1,8-ANS fluorescence changes in response to increasing peptoid concentrations were also monitored for **1–5** in the presence of these solvent additives (see Supplementary Material). 1,8-ANS emission intensity exhibited minimal change over the concentration range evaluated in guanidinium hydrochloride for all peptoids, consistent with our previous observations for **1** (Fuller et al., 2018a). Given the high observed molecular weights from the SEC experiments in guanidinium hydrochloride buffer, we attributed the low emission to the formation of more well-ordered aggregates. We reasoned that more ordering would better sequester the hydrophobic side chains from the dye. In ammonium sulfate solutions of all peptoids, however, 1,8-ANS fluorescence intensity was enhanced relative to TBS buffer. This observation suggested that peptoid aggregates formed in this solvent had more hydrophobic surface that was accessible to the dye, either solvent-exposed or in sequestered cores.

**Figure 7:**
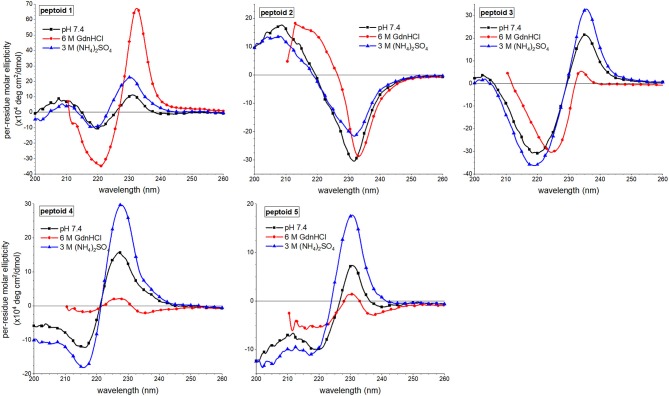
Comparisons of far-UV circular dichroism (CD) spectra of **1–5** in three different aqueous buffers. Peptoids were 50 μM in the indicated buffer, and spectra were acquired at 20°C.

Changes to the CD spectra of **1–5** in response to solvent additives revealed a unique solution behavior of **1** (Figure 7). As previously reported, the far-UV ECCD signal intensity of **1** was increased in the presence of either additive (Fuller et al., 2018a,b). In the guanidinium hydrochloride solution, however, the signal was enhanced dramatically, and we have correlated this with strong ordering of the naphthalenes in the side chains. By contrast, solvent additives had more limited effects on the spectral features of non-amphiphilic peptoid **2**. This was unsurprising given that there is little ordering of the naphthalenes in this structure. For peptoid **3**, ECCD signal intensity increased in the presence of ammonium sulfate, consistent with enhanced structural ordering of the naphthalene side chains in this solvent. In guanidinium hydrochloride, however, ECCD was minimized, suggesting that this additive induced a conformational rearrangement similar to the structure of **3** at elevated temperatures. We reasoned that the increased conformational flexibility of this peptoid allowed it to adopt a more extended conformation that oriented the naphthalenes farther from one another. Interestingly, peptoids **4** and **5** also showed enhanced ECCD signal intensities in ammonium sulfate, but minimal ECCD in guanidinium hydrochloride buffer. Again, we attributed the loss of ECCD in the guanidinium hydrochloride buffer to a conformational change that placed *N*s1npe side chains in **4** and **5** farther from one another. Changes in the near-UV region of the spectra upon changing solvent conditions were minimal (Supplementary Material).

**Figure 8:**
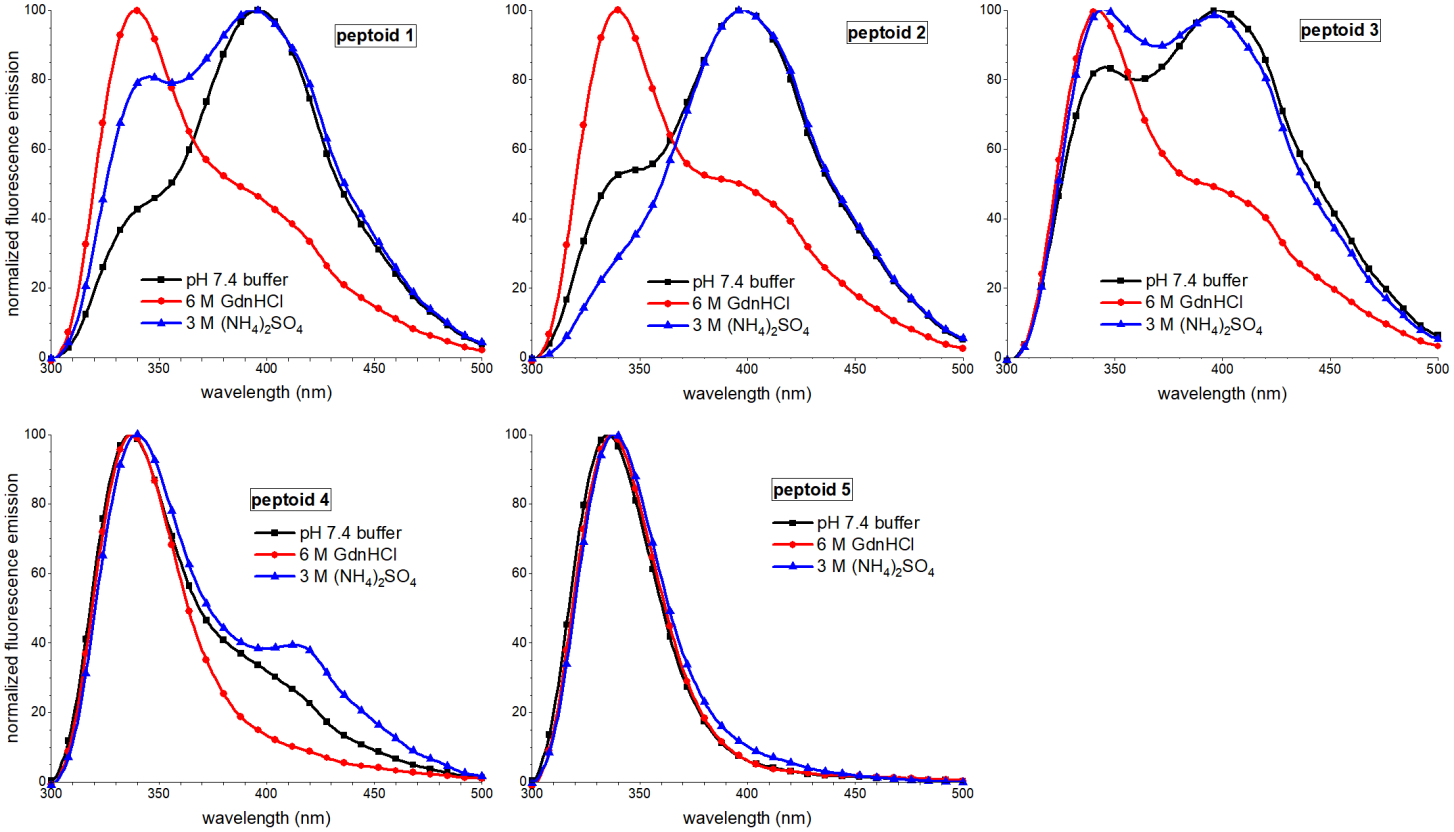
Comparisons of fluorescence emission spectra of **1–5** in three different aqueous buffers. Peptoids were 40 μM in the indicated buffer, and spectra were acquired at room temperature.

Fluorescence emission spectral features of peptoids **1–4** were also sensitive to solvent additives, though spectra of **5** were similar under all conditions (Figure 8). The most notable trend was that the excimer peak intensity was lowest when the peptoids were in the 6 M guanidinium hydrochloride buffer. The lower intensity of the excimer peak in **1** was attributed to more regular ordering of the naphthalenes in this condition, rather than to a decrease in self-association (Fuller et al., 2018a). In contrast, the lower excimer emission in **2** and **3** in guanidinium hydrochloride buffer was correlated with these peptoids' lower degree of assembly in SEC experiments. Because **4** showed a high degree of assembly in the SEC experiment run in guanidinium hydrochloride buffer, we speculated that it also could form aggregates that arranged intermolecular napthalenes farther than 4 Å. The effects of ammonium sulfate additives on peptoids' fluorescence spectral features were less consistent; excimer/monomer ratio increased for **2** and **4** but decreased for **1** and **3**; these observations were challenging to interpret.

Taken together, our observations of the solvent dependence of peptoids' solution behavior allowed us to speculate that both solvent additives, guanidinium hydrochloride and ammonium sulfate, increase peptoid self-association. However, the ordering of the aggregate changes between these conditions. In the presence of kosmotrope (ammonium sulfate), the peptoid aggregates had lower solubility and displayed more hydrophobic surface to interact with 1,8-ANS, suggesting that they were less well-ordered. The ordering of the aggregate structure appeared to be low despite the ECCD evidence for ordered chromophores in the secondary structures of **1**, **3**, **4**, and **5**. In the presence of chaotrope additive (guanidinium hydrochloride), self-association of peptoids **1–4** is also supported by SEC experiments, but a more well-ordered aggregate structure is consistent with low 1,8-ANS emission and low peptoid excimer emission. Additionally, **3**, **4**, and **5** likely form aggregates of peptoids with different secondary structures that dominate the conformational ensemble under other conditions.

## Conclusions

The results reported here confirm that peptoid sequence changes that affect amphiphilicity, conformational flexibility, and hydrophobicity greatly influence peptoid structure and peptoid self-association in aqueous solutions. We have studied self-associating amphiphilic helix peptoid **1** in depth. By disrupting the 3-fold sequence periodicity of **1**, we prepared peptoid **2** which exhibited predictably lower ordering of the naphthalene chromophores by comparison to **1**. The less regular display of hydrophobic residues likely contributed to the formation of large, disorganized aggregates of **2** that were insoluble at high concentrations. We successfully tuned peptoid conformational flexibility by substituting strongly helix-promoting *N*s1npe residues with residues that less strongly favor the *cis*-amide bond configuration, *N*s2npe and *N*spe. Despite their likely higher level of conformational heterogeneity, **3**, **4**, and **5** all showed some naphthalene chromophore ordering in their secondary structures. We speculated that the added conformational flexibility of **3** compared to **1** also allowed it to self-associate into larger aggregates than those observed for **1**. The self-association of **4** and **5** was more challenging to evaluate. Nonetheless, there is some evidence that **4** can self-associate in aqueous solution, despite being less hydrophobic than **1–3**. The influence of hydrophobicity on peptoid self-association will be the subject of an ongoing study.

Further, we have demonstrated that chaotrope and kosmotrope solution additives modulate both peptoids' secondary structures and the organization of their aggregated states. Our data suggested that higher structural homogeneity may correlate with larger, disordered aggregates, as in the case for peptoids in ammonium sulfate solution. However, secondary structural homogeneity and apparent aggregate size and organization were not correlated as strongly in the presence of chaotrope. Future studies will continue to explore the origins of these complex relationships.

Based on the observations here, we suggest that the solution behavior of **1** is unique from its isomers and close variants **2–5**. We speculate that the *N*s1npe residues confer both a level of conformational homogeneity and hydrophobicity that are important for the formation of self-assembled aggregates. Unsurprisingly, the amphiphilicity of the sequence is also a key influence on self-association. These findings suggest that design and study of new peptoids that replicate these features are most likely to yield structures with self-association features.

Ongoing studies will explore the effects of new residues on peptoid structure and self-association in aqueous solution, including other hydrophobic residues and residues that adjust peptoid net charge. Coupled with the results reported here, we will aim to articulate principles for the design of more well-structured, self-associated peptoids. Identification of new peptoids with regular, higher-order structure will expand the functional repertoire of this important class of foldamer.

## Data Availability Statement

The raw data supporting the conclusions of this article will be made available by the authors, without undue reservation, to any qualified researcher.

## Author Contributions

AF conceived and designed the experiments, carried out some of the experimental work, analyzed all of the data, and wrote the paper with input from all co-authors. CJ, EM, JM, AC, KD, JH, KM, and AO carried out the synthesis, purification, and spectroscopic study of peptoids. All authors contributed to editing of the manuscript and approved the submitted version.

## Conflict of Interest

The authors declare that the research was conducted in the absence of any commercial or financial relationships that could be construed as a potential conflict of interest.
