# Supplementary material for: Sequence Changes Modulate Peptoid Self-Association in Water
Source: Front Chem. 2020 Apr 23;8:260. doi: 10.3389/fchem.2020.00260 (PMC7191062; doi:10.3389/fchem.2020.00260)
Supplement: Supplementary file 1 [file Data_Sheet_1.PDF]

Supplementary Material

Sequence changes modulate peptoid self-association in water

Amelia A. Fuller*, Christian J. Jimenez, Ella K. Martinetto, Jose L. Moreno Jr., Anna L. Calkins, Kalli M. Dowell, Jonathan Huber, Kyra N. McComas, Alberto Ortega

Santa Clara University, Department of Chemistry & Biochemistry, Santa Clara, CA, USA

*** Correspondence:**
Amelia A. Fuller
aafuller@scu.edu

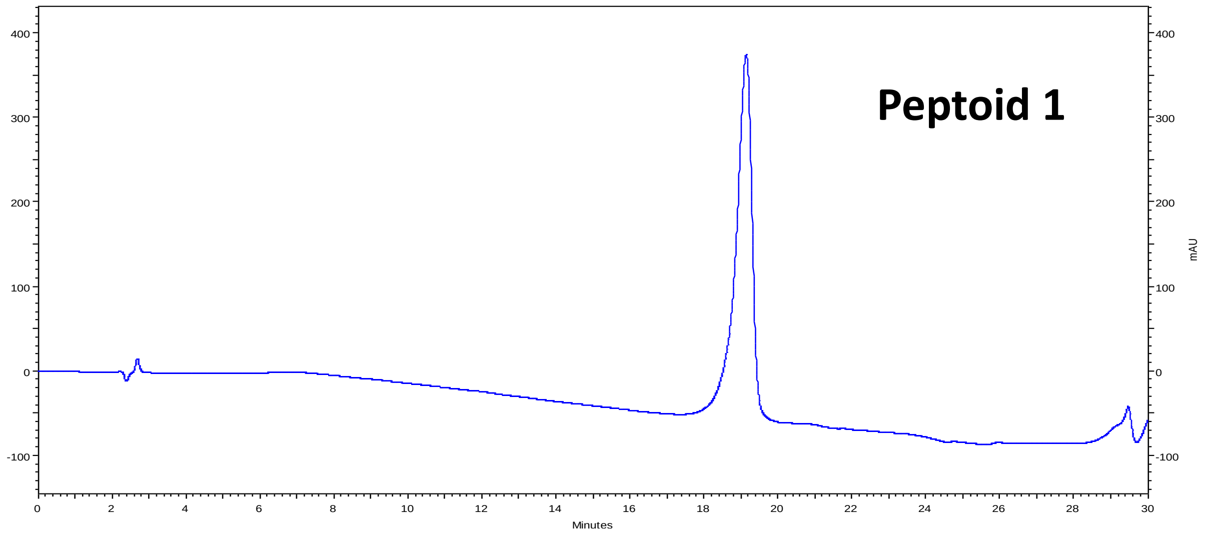
Peptoid 1
400
300
200
100
0
-100
mAU
0 2 4 6 8 10 12 14 16 18 20 22 24 26 28 30
Minutes

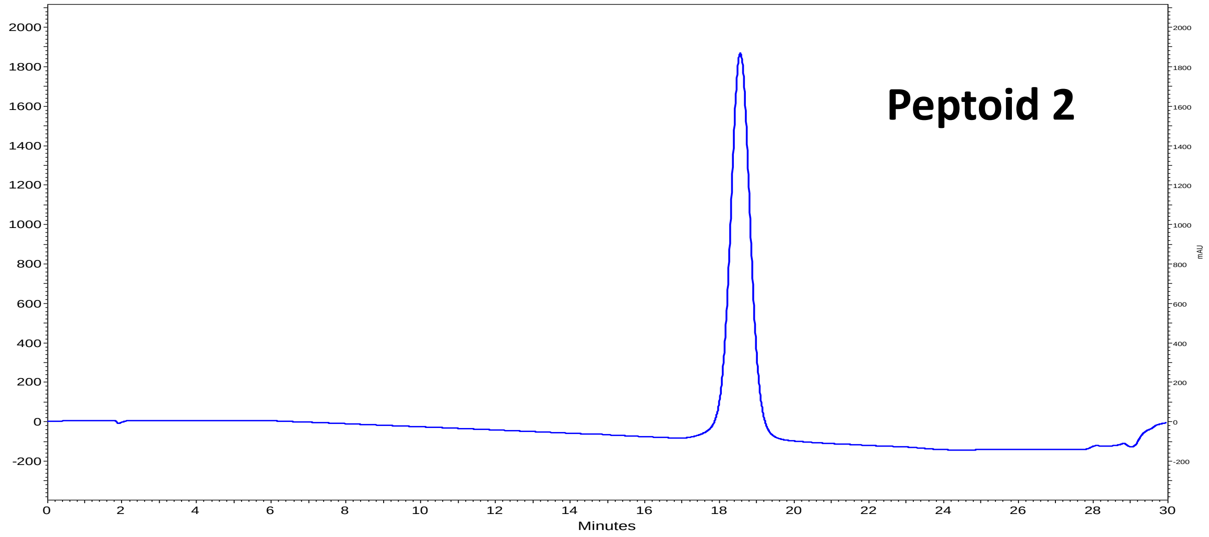
Peptoid 2
2000
1800
1600
1400
1200
1000
800
600
400
200
0
-200
mAU
0 2 4 6 8 10 12 14 16 18 20 22 24 26 28 30
Minutes

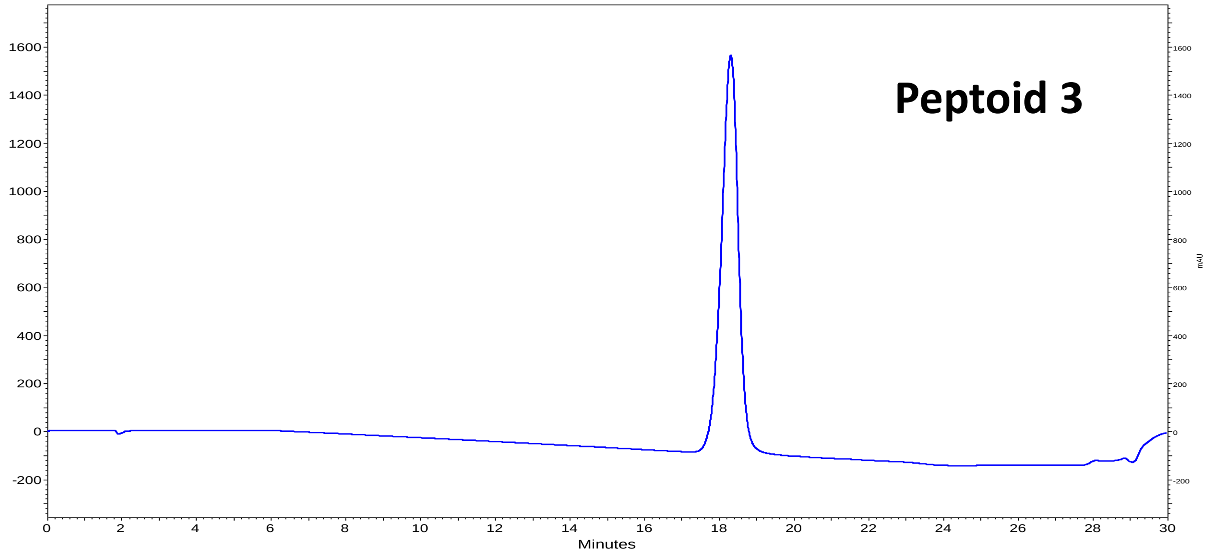
Peptoid 3
1600
1400
1200
1000
800
600
400
200
0
-200
mAU
0 2 4 6 8 10 12 14 16 18 20 22 24 26 28 30
Minutes

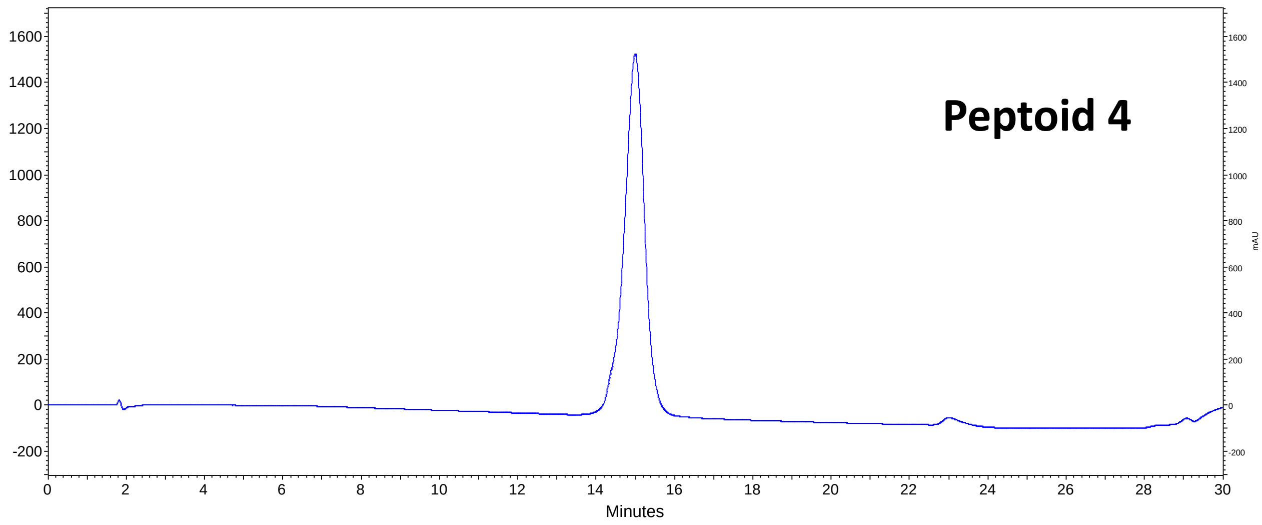

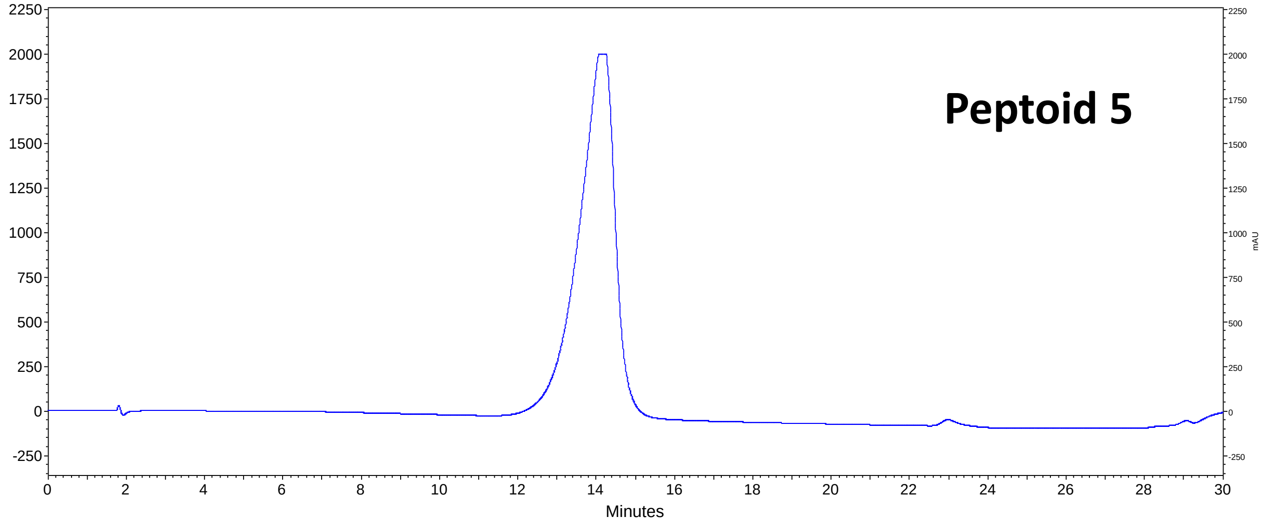

Supplementary Figure 1. Analytical HPLC chromatograms of **1-5.** Purified peptoids were eluted from an AAPPTec Spirit Peptide C18 column (5 µM, 0.46 cm x 15 cm) using a linear gradient of 5% to 95% methanol (solvent B) in 0.1% aqueous TFA (solvent A) at 0.75 mL/min flow rate. Peaks eluted were detected by absorbance at 220 nm, and data were visualized with EZChrom software

Supplementary Table 1. Calculated and observed ions for peptoids **1-5** using HRMS analysis.

Peptoid	Calculated	Observed
**1**	**[M + 2H]$^{2+}$** = 1110.036	**[M + 2H]$^{2+}$** = 1110.033
**2**	**[M + 2H]$^{2+}$** = 1110.036	**[M + 2H]$^{2+}$** = 1110.039
**3**	**[M + 2H]$^{2+}$** = 1110.036	**[M + 2H]$^{2+}$** = 1110.040
**4**	**[M + 2H]$^{2+}$** = 1060.020	**[M + 2H]$^{2+}$** = 1060.522
**5**	**[M + 2H]$^{2+}$** = 1035.012	**[M + 2H]$^{2+}$** = 1035.514

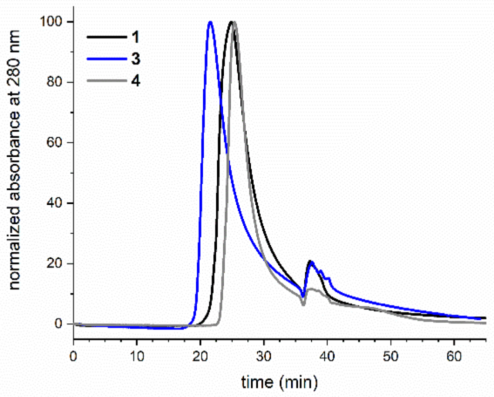

Supplementary Figure 2. SEC chromatograms of **1**, **3**, and **4** in TBS buffer. Each chromatogram was normalized to its highest intensity peak.

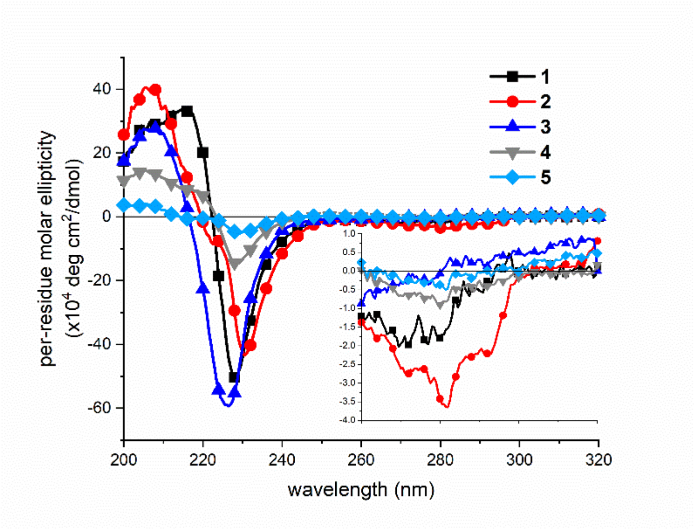

Supplementary Figure 3. CD spectra of peptoids **1-5** in methanol. Peptoid solutions were 40 μM, and spectra were acquired at 20 ºC.

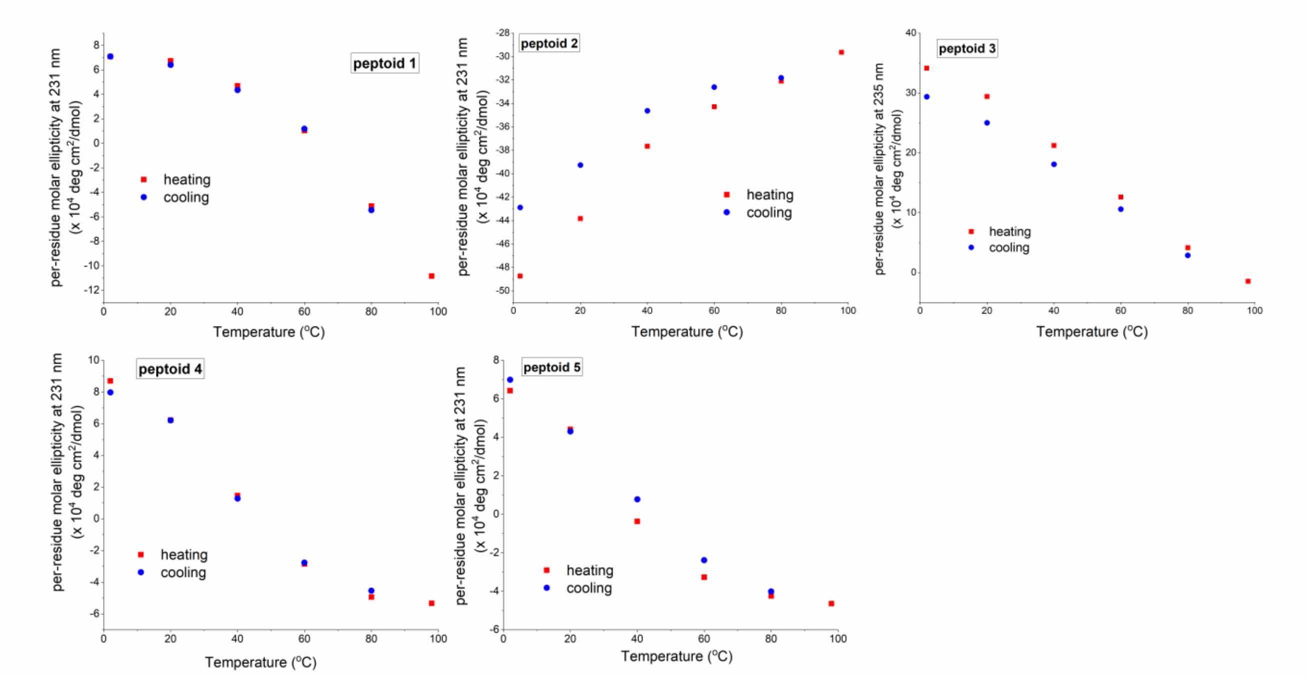

Supplementary Figure 4. CD spectral changes in response to temperature for peptoids **1-5**.

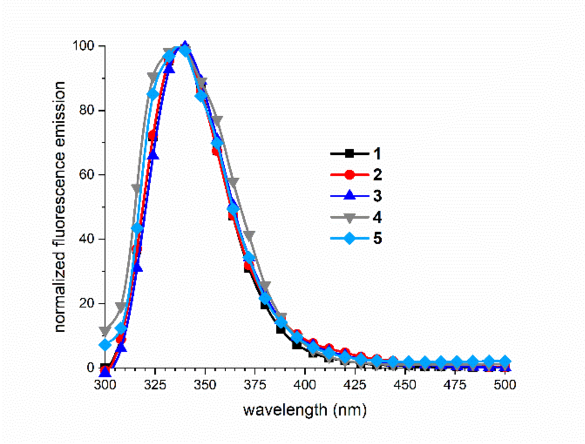

Supplementary Figure 5. Fluorescence spectra of **1-5** in methanol. Peptoid solutions were 40 µM, and each spectrum was normalized to its λ_{max}.

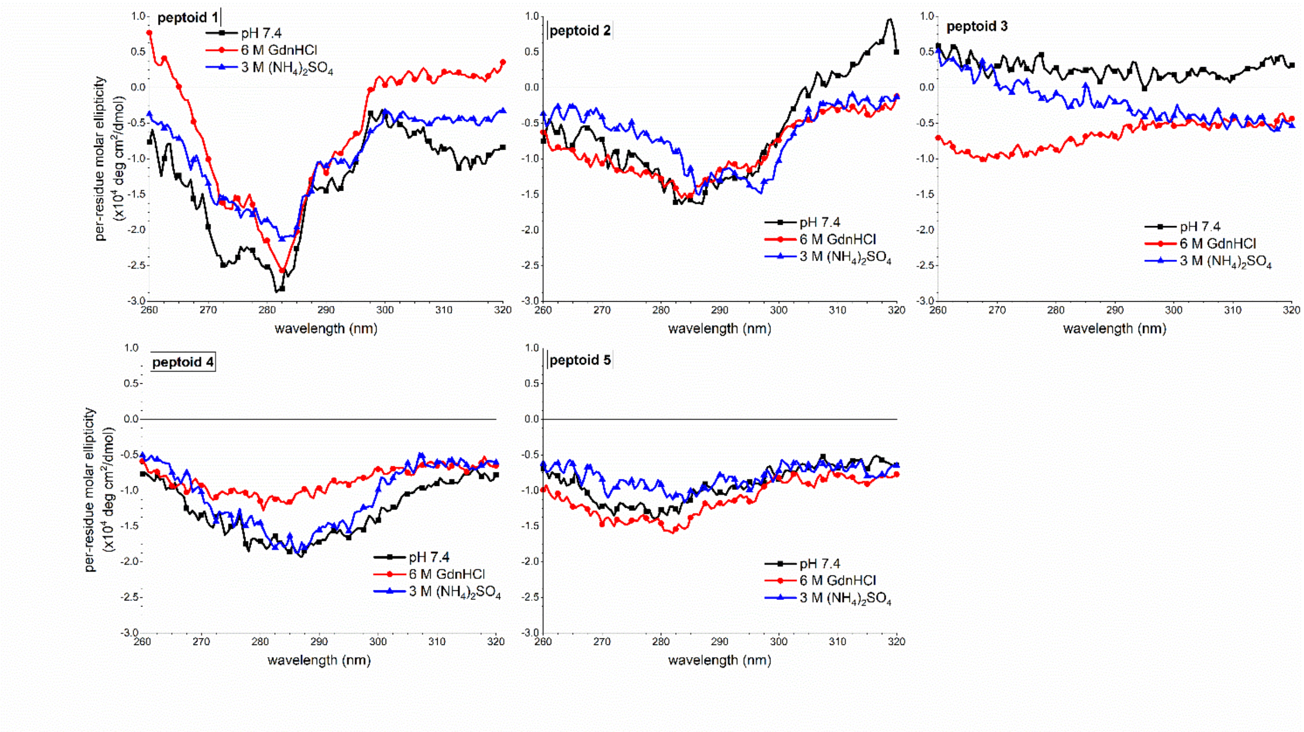

Supplementary Figure 6. Near-UV CD spectra of **1-5** in varied solvents. Peptoid solutions were 50 µM, and spectra were recorded at 20 ºC. Y-axis scales are identical in all plots.

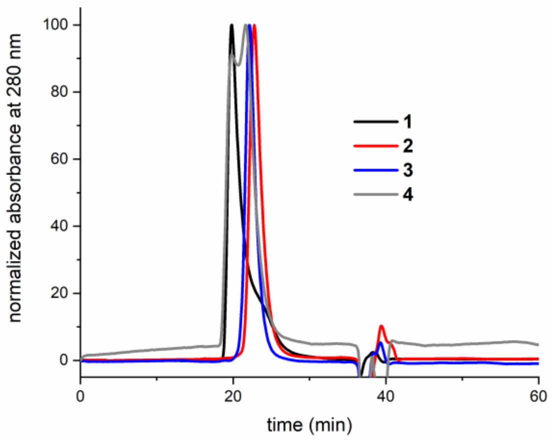

Supplementary Figure 7. SEC chromatograms for **1-4** in 6 M guandinium hydrochloride buffer. Chromatograms were normalized to the highest intensity peak.

Peptoid	Observed molecular weight	Degree of assembly
**1**	11216.28	5.0
**2**	7017.771	3.2
**3**	7816.998	3.5
**4**	8469.153	4.0

Supplementary Table 2. Observed molecular weights and degrees of assembly for peptoids analyzed by SEC in 10 mM phosphate + 6 M guanidinium hydrochloride buffer.

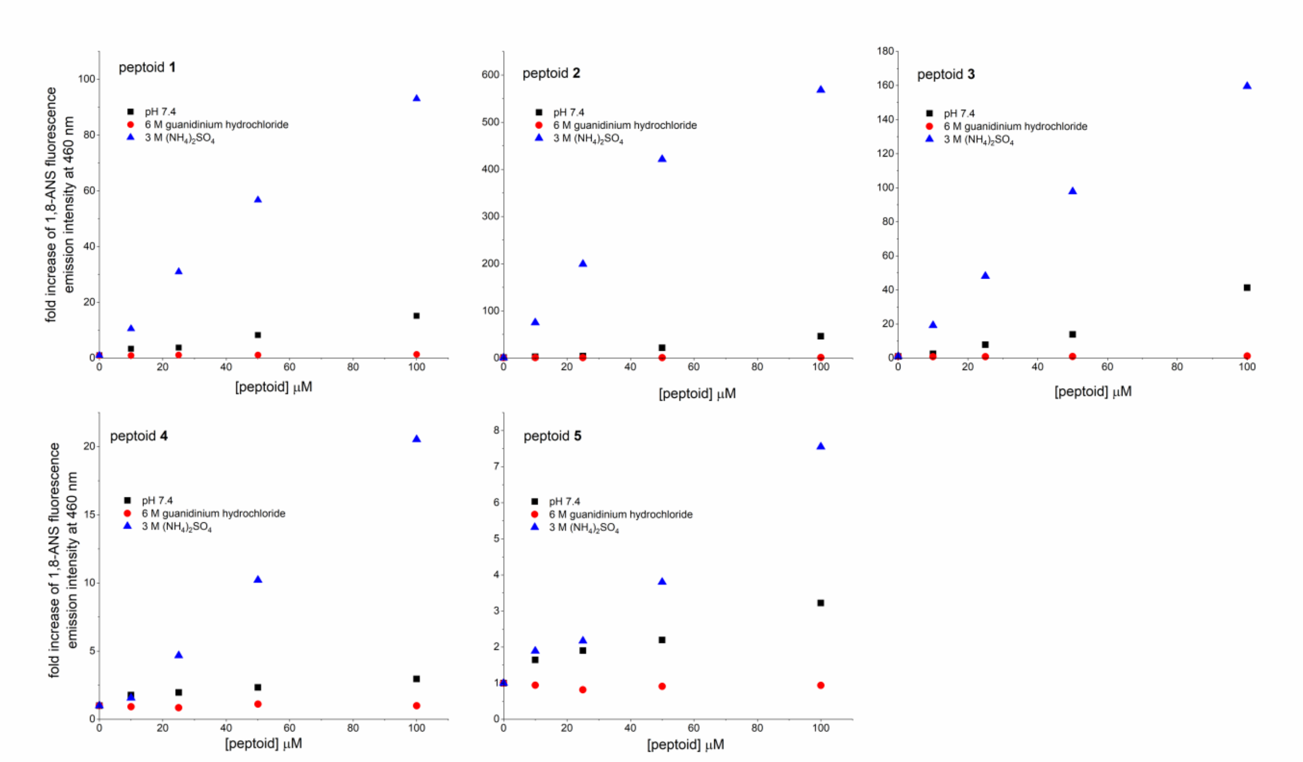

Supplementary Figure 8. Changes to 1,8-ANS fluorescence emission intensity in varied solvents with increasing concentrations of **1-5**. Note that y-axis scales vary for different peptoids.
